# The dissociable effects of reward on sequential motor behavior

**DOI:** 10.1152/jn.00467.2021

**Published:** 2022-06-01

**Authors:** Sebastian Sporn, Xiuli Chen, Joseph M. Galea

**Affiliations:** ^1^School of Psychology and Centre for Human Brain Health, University of Birmingham, Birmingham, United Kingdom; ^2^Department of Clinical and Movement Neuroscience, Queens Square Institute of Neurology, University College London, London, United Kingdom.

**Keywords:** complex sequential motor behavior, motor fusion, motor learning, movement fusion, reward

## Abstract

Reward has consistently been shown to enhance motor behavior; however, its beneficial effects appear to be largely unspecific. For example, reward is associated with both rapid and training-dependent improvements in performance, with a mechanistic account of these effects currently lacking. Here we tested the hypothesis that these distinct reward-based improvements are driven by dissociable reward types: monetary incentive and performance feedback. Whereas performance feedback provides information on how well a motor task has been completed (knowledge of performance), monetary incentive increases the motivation to perform optimally without providing a performance-based learning signal. *Experiment 1* showed that groups who received monetary incentive rapidly improved movement times (MTs), using a novel sequential reaching task. In contrast, only groups with correct performance-based feedback showed learning-related improvements. Importantly, pairing both maximized MT performance gains and accelerated movement fusion. Fusion describes an optimization process during which neighboring sequential movements blend together to form singular actions. Results from *experiment 2* served as a replication and showed that fusion led to enhanced performance speed while also improving movement efficiency through increased smoothness. Finally, *experiment 3* showed that these improvements in performance persist for 24 h even without reward availability. This highlights the dissociable impact of monetary incentive and performance feedback, with their combination maximizing performance gains and leading to stable improvements in the speed and efficiency of sequential actions.

**NEW & NOTEWORTHY** Our work provides a mechanistic framework for how reward influences motor behavior. Specifically, we show that rapid improvements in speed and accuracy are driven by reward presented in the form of money, whereas knowledge of performance through performance feedback leads to training-based improvements. Importantly, combining both maximized performance gains and led to improvements in movement quality through fusion, which describes an optimization process during which sequential movements blend into a single action.

## Graphical abstract

**Figure:**
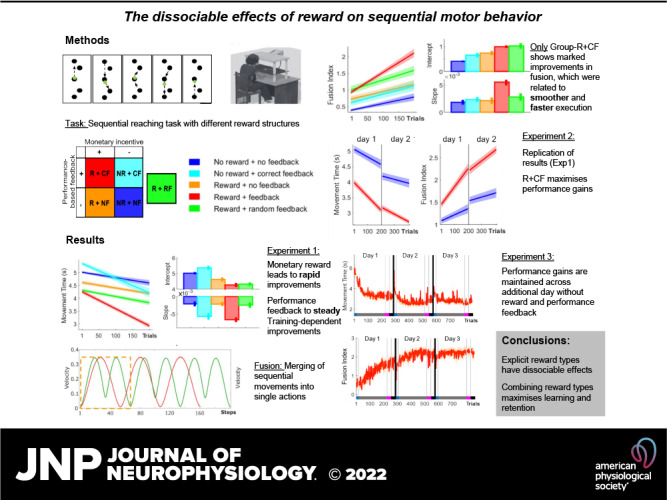

## INTRODUCTION

Research into the effects of reward on motor behavior has consistently shown that reward enhances performance (1–11). Consequently, reward, as a tool to shape motor behavior, has gained much scientific interest, particularly with regard to its strategic and beneficial use in rehabilitation. However, the beneficial effects of reward on behavior appear to be largely unspecific. Whereas studies using saccadic or discrete reaching movements have consistently found that reward rapidly improved the speed-accuracy function (i.e., transient improvement within a single trial) (1, 3–5, 11–14), research employing force and button-press tasks showed reward-related improvements in learning and/or retention (i.e., improvements across trials) (8–10, 15–17). Therefore, a mechanistic account for how reward enhances motor performance is lacking, which restricts the potential of a targeted use of reward in clinical settings. Crucially, such a mechanistic account will have to be able to account for both the invigoration and training-dependent learning effects associated with reward.

An interesting possibility is that these distinct reward-based improvements are driven by dissociable reward types. Reward is most commonly provided through a monetary incentive that is presented as trial-based performance feedback (4, 8–10, 15). Therefore, performance feedback is coupled with a monetary incentive (e.g., 5 points correspond to earning £0.05). However, both represent a form of explicit reward, and it is not clear whether they influence motor behavior in a similar manner. Performance feedback represents a reinforcement-based teaching signal (i.e., reward-prediction error) (18, 19) that provides information on how well a motor task has been completed (knowledge of performance) and has been shown to enhance other forms of motor learning (11, 20–24). In contrast, explicit reward presented via a monetary incentive increases the motivation to perform optimally without necessarily providing a performance-based learning signal (25). Recent research decoupled monetary incentive from performance feedback and found that although performance feedback alone was not sufficient to induce skill leaning in a pinch force reproduction task, combining it with a monetary reward was (15). However, the effect of a monetary incentive alone on motor behavior was not accounted for (15). Therefore, to dissociate the effects of both on motor behavior it is crucial to systematically assess them in isolation and in combination.

To this end, we designed a novel complex sequential motor task in which participants were asked to complete a continuous sequence of eight reaching movements. Participants received a combination of both explicit rewards (i.e., money and performance feedback), which allowed us to systematically evaluate how they influence performance during a complex sequential reaching task. We hypothesized that explicit reward presented via a monetary incentive will lead to rapid improvements in performance during early training. In contrast, accurate performance feedback will lead to learning-related improvements across training. Importantly, in line with recent findings, we hypothesized that combining monetary incentive with accurate performance feedback will maximize performance gains (15).

*Experiment 1* confirmed that monetary incentive and performance feedback have dissociable effects on motor behavior. Specifically, participants who received a monetary incentive, irrespective of the availability and quality of performance-based feedback, rapidly reduced movement times (MTs) during early training. Additionally, performance feedback led to training-related improvements in MT irrespective of reward availability. Importantly, this was only the case when performance feedback was accurate. No training-related improvements were observable when the feedback was random. Crucially, combining monetary incentive with accurate performance feedback resulted in both a rapid reduction and a learning-related improvement in MT that maximized performance gains. Further analysis revealed that these performance gains were associated to movement fusion. Fusion describes an optimization process during which individual motor elements are blended into a combined singular action (26–28). Therefore, movement fusion represents an effective strategy to achieve quicker MTs by producing fast reaching movements while simultaneously reducing dwell times when transitioning between reaches. *Experiment 2* provided a replication in which the combination of monetary incentive and accurate performance feedback improved MTs across 2 days of training and led to a substantial increase in movement fusion. Critically, movement fusion was associated with increases in movement smoothness, which also reflected the predictions of a model that optimized jerk across the sequential movement (29, 30). These results suggest that performance became more energetically efficient (31, 32), which may explain the results from *experiment 3*, where improvements in performance persisted for 24 h even when reward was no longer available.

## METHODS

### Participants

One hundred twenty-one participants (24 males; age range 18–35 yr) were recruited to participate in three experiments, which had been approved by the local research ethics committee of the University of Birmingham. All participants were novices to the task paradigm and by questionnaire were self-reportedly free of any motor, visual, and cognitive impairment. Most participants were self-reportedly right-handed (*N* = 9 left-handed participants) and gave written informed consent before the start of the experiment. For their participation, participants were remunerated with either course credits or money (£7.50/h) and were able to earn additional money during the task depending on their performance. Depending on the experiment, participants were pseudorandomly allocated to one of the available groups.

### Experimental Apparatus

**Figure 1.**
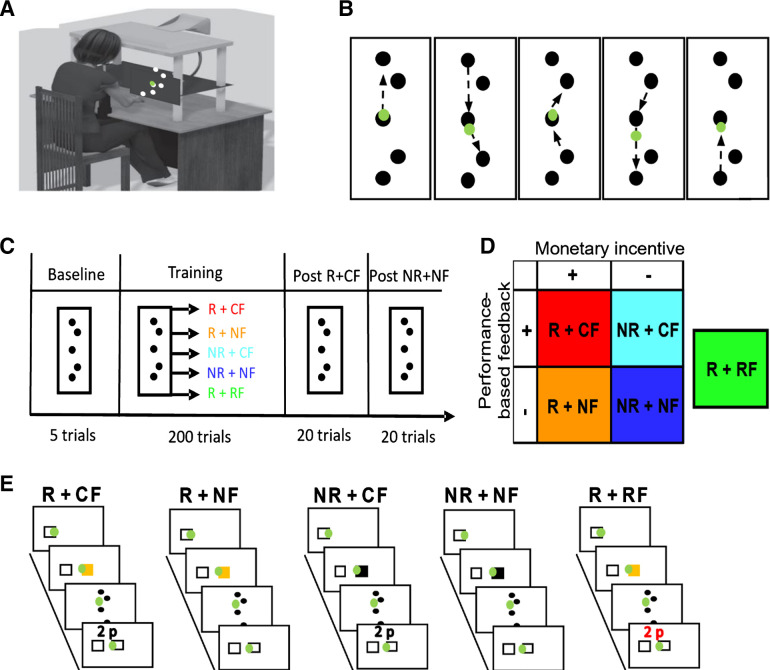
Experimental setup. *A*: participants wore a motion-tracking device on the index finger, and the unseen reaching movements were performed across a table while a green cursor matching the position of index finger was viewable on a screen. *B*: 8-movement sequential reaching task. Participants started from the center target. *C*: study design. Before the start of the experiment, participants were trained on the reaching sequence and were then asked to perform 5 baseline trials. Randomly allocated to 1 of 5 groups, participants completed 200 trials during training and an additional 20 trials in each postassessment; 1 with reward (post-R+CF) and 1 without (post-NR+NF) (counterbalanced across participants). *D*: groups. The feedback regime differed with regard to the availability of both monetary incentive and performance-based feedback. Participants either received monetary incentive (R) or not (NR). Similarly, participants were provided with correct (CF), no (NF), or random (RF) performance-based feedback. *E*: monetary incentive trials were cued with a visual stimulus (yellow start box) before the start of the trial (Group_-R+CF_, Group_-R+NF_, and Group_-R+RF_). In contrast, in trials without monetary incentive no visual stimulus was provided (black start box) and participants were instructed to be as fast and accurate as possible. Feedback was provided after a completed trial but only in groups who received feedback (Group_-R+CF_, Group_-R+NF_, and Group_-R+RF_).

All experiments were performed with a Polhemus 3SPACE FASTRAK tracking device (Colchester, VT; with a sampling rate of 110 Hz). Participants were seated in front of the experimental apparatus, which included a table, a horizontally placed mirror 25 cm above the table, and a screen (Fig. 1*A*). A low-latency Apple Cinema screen was placed 25 cm above the mirror and displayed the workspace and participants’ hand position (represented by a green cursor, diameter 1 cm). On the table, participants were asked to perform two-dimensional (2-D) reaching movements. Looking into the mirror, they were able to see the representation of their hand position reflected from the screen above. This setup effectively blocked their hand from sight. The experiment was run with MATLAB (The MathWorks, Natick, MA) with Psychophysics Toolbox 3.

### Task Design

Participants were asked to hit a series of targets displayed on the screen (Fig. 1*B*). Four circular (1-cm diameter) targets were arranged around a center target (“via target”). Starting in the via target, participants had to perform eight continuous reaching movements to complete a trial. *Targets 1* and *4* were displaced by 10 cm on the *y*-axis, whereas *targets 2* and *3* were 5 cm away from the via target with an angle of 126° between them (Fig. 1*B*). To start each trial, participants had to pass their cursor though the preparation box (2 × 2 cm) on the left side of the workspace, which triggered the appearance of the start box (2 × 2 cm) in the center of the screen. After moving the cursor into the start box, participants had to wait for 1.5 s for the targets to appear. This ensured that participants were stationary before reaching for the first target. Target appearance served as the go signal, and the start box turned into the via target (circle). Upon reaching the last target (via target), all targets disappeared, and participants had to wait for 1.5 s before being allowed to exit the start box to reach for the preparation box to initiate a new trial. Participants had to repeat a trial if they missed a target or performed the reaching order incorrectly. Similarly, exiting the start box too early either at the beginning or at the end of each trial resulted in a missed trial.

### Reward Structure and Feedback

In *experiment 1* (*N* = 74), participants were randomly allocated to one of five groups: *1*) no reward and no performance feedback (Group_-NR+NF_; *N* = 15), *2*) no reward and accurate performance feedback (Group_-NR+CF_; *N* = 15), *3*) monetary incentive and no performance feedback (Group_-R+NF_; *N* = 15), *4*) monetary incentive and accurate performance feedback (Group_-R+CF_; *N* = 15), and *5*) monetary incentive and random performance feedback (Group_-R+RF_; *N* = 14; Fig. 1, *C–E*). This design allowed us to systematically evaluate how monetary incentive and performance feedback influence performance during a complex sequential reaching task.

Participants who received monetary incentive were informed that faster MTs would earn them more money. Reward trials were cued with a visual stimulus before the start of the trial (Fig. 1*E*). Once participants moved into the preparation box, the start box appeared in yellow (visual stimulus). In contrast, participants who were in a NR group were told to move as fast and accurately as possible, and here the start box remained black. Performance feedback was provided after completion of a trial while participants moved from the start box to the preparation box to initiate a new trial. Feedback was displayed on the top of the screen (i.e., “2p out of 5p”). We used a closed-loop design to calculate the feedback in each trial. To calculate this, we included the MT values of the last 20 trials and organized them from fastest to slowest to determine the rank of the current trial within the given array. A rank in the top three (≥90%) returned a value of 5p, ranks ≥ 80% and < 90% were valued at 4p; ranks ≥ 60% and < 80% were awarded 3p; ranks ≥ 40% and < 60% earned 2p; and 1p was awarded for ranks ≥ 20% and < 40%. A rank in the bottom three (<20%) returned a value of 0p. When participants started a new experimental block, performance in the first trial was compared to the last 20 trials of the previously completed block. Whereas participants in Group_-R+CF_ and Group_-R+RF_ were told that the performance feedback corresponds to money (i.e., 5p = 5 pence), Group_-NR+CF_ was informed that it refers to points (i.e., 5p = 5 points) that do not add money. In contrast to the CF groups, Group_-R+RF_ received random feedback, which was not performance based but was drawn randomly from feedback given to participants in Group_-R+CF_. To this end, we strung together all reward values given to participants in this group and randomly chose a value for feedback in a given trial for Group_-R+RF_. Participants therefore received feedback that was similar in reward probability without corresponding to actual performance.

### *Experiment 1* Experimental Procedure

In this experiment, we investigated whether monetary incentive and performance feedback have dissociable effects on a sequential reaching task. The experiment included an initial learning phase before the start of the experiment as well as a Baseline, training, and two postassessments. Participants were pseudorandomly allocated to one of the five groups (*N* = 74; Fig. 1, *C* and *D*).

#### Learning.

We included a learning phase before the start of the experiment for participants to be able to memorize the reaching sequence. This allowed us to attribute any performance gains to improvements in execution rather than memory. Once participants waited 1.5 s inside the start box, the targets appeared, which were numbered clockwise from 1 to 4 starting with the central top target. Participants were also able to see a number sequence at the top left of the screen displaying the order of target reaches (1–3–2–4). Participants were instructed to hit the targets according to the number sequence while also hitting the via target in between target reaches. They had to repeat a trial if they missed a target or performed the reaching order incorrectly. Similarly, exiting the start box too early either at the beginning or at the end of each trial resulted in a missed trial. After a cued trial, participants were asked to complete a trial from memory without the number sequence or numbers inside the targets. If participants failed a no-cue trial more than twice, cues appeared in the following trial as a reminder. After a maximum of 10 cue and 10 no-cue trials, participants completed this block.

#### Baseline.

Participants in both groups completed 10 Baseline trials, which were used to assess whether there were any pretraining differences between groups. All groups were instructed to “move as fast and accurately as possible,” whereas no monetary incentive or performance-based feedback was available.

#### Training.

Participants completed 200 training trials and received a combination of monetary incentive and performance feedback depending on the group that they were assigned to. The monetary incentive groups were informed that during this part they would be able to earn money depending on how fast they completed each trial (200 reward trials).

#### Postassessments.

After training, participants from all groups were asked to complete two postassessments (20 trials each), one with both monetary incentive and accurate performance feedback available (post-R+CF) and one without either (post-NR+NF). The order was counterbalanced across participants.

### *Experiment 2* Experimental Procedure

In this experiment (*N* = 42), we aimed to partially replicate the results from *experiment 1*. In *experiment 2*, only Group_-R+CF_ and Group_-NR+NF_ were included, to contrast the most beneficial feedback regime (i.e., monetary incentive with accurate performance feedback) with its logical opposite. Additionally, we added a further testing day (*day 2*) to investigate whether movement fusion can be further enhanced with additional training. During *day 2*, participants underwent the same experimental protocol as *day 1* (Fig. 1*C*).

### *Experiment 3* Experimental Procedure

In this experiment, we aimed to test how robust reward-driven performance gains were over an additional testing day without monetary incentive and performance feedback. Participants (*N* = 5) underwent the same regime as the reward group in *experiment 2* on the first 2 testing days. On the third testing day after baseline, participants were asked to complete 200 NR+NF trials.

### Data Analysis

Analysis code is available on the Open Science Framework website, alongside the experimental data sets, at https://osf.io/62wcz/. The analyses were performed in MATLAB (The MathWorks, Natick, MA).

### Movement Time

Movement time (MT) was measured as the time between exiting the start box and reaching the last target. This excludes reaction time, which describes the time between target appearance and when the participants’ start position exceeded 2 cm. In summary, monetary incentive and performance feedback have dissociable effects on motor behavior. Importantly, pairing both maximized performance gains and accelerated the slow optimization process of movement fusion, which leads to stable improvements in the speed and efficiency of sequential actions.

### Maximum and Minimum Velocity

Through the derivative of positional data (*x, y*), we obtained velocity profiles for each trial, which were smoothed with a Gaussian smoothing kernel (σ = 2). The velocity profile was then divided into segments representing movements to each individual target (8 segments) by identifying when the positional data was within 2 cm of a target. We measured the maximum velocity (*v*_max_) of each segment by finding the maximum velocity:

(*1*) $$v_{\max}\;≜{\text{ max}}_{\epsilon \;\left[t_{1}\;t_{2}\right]}\;v(t)$$

where *v*(*t*) is the velocity of segment *t* and *t*_1_ and *t*_2_ represent the start and end of segment *t*, respectively. Similarly, minimum velocity (*v*_min_) was determined by measuring the minimum velocity when participants were inside a target (7 targets), using:

(*2*) $$v_{\min}\;≜\;\mathrm{mi}n_{\epsilon \;\left[t_{1}\;t_{2}\right]}\;v(t)$$

The individual maximum and minimum velocities were then averaged for each trial.

### Fusion Index

Fusion describes the blending together of individual motor elements into a singular smooth action. This is represented in the velocity profile by the stop period between the two movements gradually disappearing and being replaced by a single velocity peak (see Fig. 4, *A* and *B*) (26–28). To measure fusion, we compared the mean maximum velocities of two sequential reaches with the minimum velocity around the via point. The smaller the difference between these values, the greater coarticulation had occurred between the two movements (see Fig. 4*B*) (33). We calculated movement fusion by

(*3*) $$\text{fusion index }≜1-\;\frac{\left(\frac{v_{\mathrm{ma}x^{1\;}}-\;v_{\mathrm{ma}x^{2\;}}}{2}\right)-\;v_{\min}}{\left(\frac{v_{\mathrm{ma}x^{1\;}}-\;v_{\mathrm{ma}x^{2\;}}}{2}\right)}$$

with $v_{{\max\;}^{1}}$ and $v_{{\max\;}^{2}}$ representing the velocity peak value of two reaching movements, respectively, and *v*_min_ representing the minimum value between these two points. We normalized the obtained difference, ranging from 0 to 1, with 1 indicating a fully coarticulated movement. Given that in this task 7 transitions had to be completed, the maximum fusion index (FI) value was 7 in each trial.

### Spatial Reorganization

In addition to FI, fusion can also be expressed spatially as the radial distance between the maximum velocity (*v*_max_) on the submovements and the minimum velocity (*v*_min_) around the via point (see Fig. 7*A*). This distance becomes smaller with increased movement fusion (26, 28) and reflects the merging of two submovements into one (Supplemental Fig. S3; all Supplemental Materials are available at https://doi.org/10.6084/m9.figshare.16831774.v1). To measure these changes in radial distance between *v*_max_ and v_min_, we used a sliding window approach of 10 trials at a time. For each target reach (excluding the first and the last) we fitted a confidence ellipse (34) with a 95% confidence criterion around the scatter of the spatial position (*x, y*) of each *v*_max_ of the included trials (see Fig. 7*A*). The confidence ellipses were obtained by using principal component analysis to determine the minimum and maximum dispersion of the included data points in the *x-y* plane. To measure the distance between the scatter and its corresponding via point, we determined the ellipse’s centroid (point of intersection of ellipse’s axes) and calculated the radial distance to the via point. The obtained distance values were normalized and ranged from 0 to 100%, with 100% representing 0-cm distance between the centroid and the via point. Considering that individual reaching movements display a bell-shaped velocity profile, with the *v*_max_ situated approximately in the center of the movement, radial distance values between 45% and 55% can be expected if each movement is executed individually (Supplemental Fig. S3).

### Minimum-Jerk Model

A traditional minimum-jerk model for motor control is guided by optimization theory, where a “cost” is minimized over the trajectory (29, 30). In the case of the minimum-jerk model, the cost is defined as the squared jerk (3rd derivative of position with respect to time):

(*4*) $$\mathrm{jerk}\;≜\;\frac{1}{2}\;\int_{t_{1}}^{t_{2}}\left({\left|\frac{d^{3}x}{dt^{3}}\;\right|}^{2}+\;{\left|\frac{d^{3}y}{dt^{3}}\;\right|}^{2}\right)\;dt$$

Here *x* and *y* represent the position of the index finger over time (*t*), and *t*_1_ and *t*_2_ define the start and end of a trial in seconds (*t*). The MATLAB code (35) provided by Todorov and Jordan (30) was used to compute the minimum-jerk trajectory (trajectory that minimized *Eq. 4*) and the accompanying velocity profile, given a set of via points, start/end position, and movement time (30). We then calculated the mean squared error (immse function in MATLAB) between the predicted and actual velocity profile, which were both normalized and interpolated (*N* = 500), to estimate the fit on a trial-by-trial basis. Because of the two-dimensional structure of trajectories, we used velocity profiles rather than the trajectories for this comparison.

### Spectral Arc Length

To further assess movement smoothness, we measured spectral arc length (see Supplemental Fig. S4). Although we decided to use the traditional jerk metric in our modeling analysis, to allow for comparisons with prior literature, spectral arc length has been shown to be less sensitive to differences in MT and more sensitive to changes in smoothness (36, 37). The spectral arc length is derived from the arc length of the power spectrum of a Fourier transformation of the velocity profile. We used an open-source MATLAB toolbox to calculate this value for each trajectory (38).

For both spectral arc length and the minimum-jerk model, we only included noncorrected trials. Trials that were classified as corrected included at least one corrective movement to hit a previously missed target. These additional movements added peaks to the velocity profile, which complicated model comparison and increased jerkiness disproportionally. Therefore, 1,820 trials were excluded for both analyses (8.68% of all trials).

### Statistical Analysis

ANOVAs (*experiment 1*) and Wilcoxon tests (*experiment 2*) were used to analyze differences in performance during baseline. To assess whether monetary incentive and performance-based feedback have distinct effects on performance during training, in *experiments 1* and *2* we computed 1,000 bootstrap estimates of the data for each group. Each estimate represented a randomly generated data set (*N* = 15, with *N* = 14 for Group_-NR+CF_) with replacement. Specifically, one participant was randomly chosen from the group pool and added to the new data set. This participant was subsequently included into the group pool again before another participant was randomly selected to be added to the new data set. Therefore, the same participant could be added to the new data set multiple times. A simple polynomial model [*f*(*x*) = *p1* × *x + p2*] was then fit to the mean of the new trial-by-trial training data set (200 trials) for each of the 1,000 bootstrap estimates of each group. The 95% confidence intervals based on a group comparison were used for each model parameter to assess significant differences between groups (39). Whereas *p2* represents the performance intercept during early training (1st to 15th trial), *p1* corresponds to the gradient (learning rate) across training. This model and analysis was chosen as it provided a simple but powerful assessment of the dissociable rapid (intercept) and learning-related effects associated with the different forms of feedback across groups.

We further aimed to assess whether changes in performance reflect “true” motor learning and whether monetary reward and/or feedback enhances motor learning. Importantly, improvements could also be driven by transient factors such as arm stiffness, cocontraction, and/or task knowledge. To this end, we conducted mixed-model ANOVAs to assess whether changes in performance from Baseline to post-NR+NF reflect motor learning [Baseline (all trials), post-NR+NF (all trials)] and group (*experiment 1*: Group_-R+CF_, Group_-R+RF_, Group_-R+NF_, Group_-NR+CF_, and Group_-NR+NF_). No monetary reward or feedback was provided during both Baseline and post-NR+NF in each group. Consequently, all groups were under the same “neutral” conditions. We hypothesized that a significant main effect for time point (Baseline vs. post-NR+NF) will provide circumstantial evidence that performance improvements across training reflect motor learning and are not solely driven by transient effects such as arm stiffness and cocontraction. Additionally, we believe that our experimental design, which includes a learning phase before the start of the main experiment, prevents improvements from being solely related to explicit factors such as task knowledge. At any rate, task knowledge should be similar across participants and groups after the learning phase. Similarly, mixed-model ANOVAs were used to assess statistical significance during the postassessments, with condition [post-R+CF, post-NR+NF (all 20 trials in each)] and group (*experiment 1*: Group_-R+CF_, Group_-R+RF_, Group_-R+NF_, Group_-NR+CF_, and Group_-NR+NF_; *experiment 2*: Group_-R+CF_ and Group_-NR+NF_) as factors. Note that that the model analysis was not required here as performance was stable across trials. We used one-sample Kolmogorov–Smirnov tests to test our data for normality and found that all measures were nonparametric. Median values were therefore used as input in all mixed-model ANOVAs (similar to Ref. 40). Wilcoxon tests were employed when a significant interaction and/or main effects were reported. The results were corrected for multiple comparisons with false discovery rate [fdr_bh(stats, ‘alpha’, 0.05) in MATLAB (41)]. Therefore, the *P* values presented in results have been adjusted to the number of comparisons conducted (comparing each group with all others, which amounts to *N* = 10). Linear partial correlations (fitlm function in MATLAB) were used to measure the degree of association between the chosen variables, while accounting for the factor group. Piecewise linear spline functions were fitted through the scatter of spatial distance values, and FI levels with least square optimization by means of shape language modeling (SLM) (42). We used three knots as input for the linear model. A repeated-measures ANOVA was used to test for significance of our results in *experiment 3*. We compared performance with time point [early training (first 15 trials), late training (last 15 trials) over all 3 testing days] as the within factor. Because our data were nonparametric after one-sample Kolmogorov–Smirnov tests, we included median values as input for all repeated-measures ANOVAs. Wilcoxon test was used as post hoc test, and multiple comparisons were corrected for with Bonferroni corrections. Repeated-measures ANOVAs were chosen to analyze performance in *experiment 3* because only Group_-R+CF_ was included, which did not require a more complex group comparison.

## RESULTS

To assess the influence of monetary incentive and performance-based feedback on complex, sequential movements we developed a novel reaching task in which participants made eight sequential reaching movements to designated targets (1 trial) with a motion tracking device (Fig. 1, *A* and *B*). Before the start of the experiment, participants were trained on the sequence without a time constraint until reaching a learning criterion of five successful trials in a row. Importantly, missing a target resulted in an immediate abortion of the current trial, which participants then had to repeat. This allowed us to focus on performance gains in speed rather than accuracy (which was high before the start of the main experiment). Participants then completed a Baseline period (10 trials) during which they were encouraged to complete each trial “as fast and as accurately as possible” (Fig. 1*C*). Afterwards, participants completed 200 training trials. During training, participants were placed under different feedback regimes that differed with regard to both the availability of monetary incentive and performance feedback (Fig. 1*D*). This allowed us to systematically evaluate how they influence performance during a complex sequential reaching task. Participants in Group_-R+CF_ (monetary incentive + correct feedback) were able to earn money depending on their movement time (MT) and received performance-based feedback [the amount of money (0–5p) awarded in a given trial] after each trial. Monetary incentive trials were cued with a yellow start box (Fig. 1*E*), and the performance feedback was calculated with a closed-loop design comparing MT performance on a given trial with performance on the last 20 trials. This provided the participants with graded feedback (0–5p) of their MT performance relative to their recent performance (see methods). In contrast, participants in Group_-R+NF_ (monetary incentive + no feedback) only received monetary incentive (yellow start box cue) and were not provided with performance feedback after each trial. Instead, participants received the accumulated monetary reward at the end of training. Similarly to Group_-R+CF_, participants in Group_-R+RF_ (monetary incentive + random feedback) received both monetary incentive and performance-based feedback. However, the performance feedback was random (see methods) and thus did not correspond to participants’ actual performance.

Finally, whereas participants in both Group_-NR+CF_ (no monetary incentive + correct feedback) and Group_-NR+NF_ (no monetary incentive + no feedback) did not receive any monetary incentive during training, performance feedback was provided in Group_-NR+CF_. However, participants were told that this feedback was not related to monetary incentive (Fig. 1*E*). After training, both groups engaged in a rewarded (post-R+CF) and a nonrewarded (post-NR+NF) postassessment (20 trials each). Therefore, all groups received both monetary incentive and correct feedback during post-R+CF, whereas neither was available during post-NR+NF, the postassessment intended to compare performance between groups when under the same condition (Fig. 1*C*).

### Monetary Incentive Led to a Rapid Decrease in MT whereas Performance-Based Feedback Was Associated with Learning-Related MT Improvements

**Figure 2.**
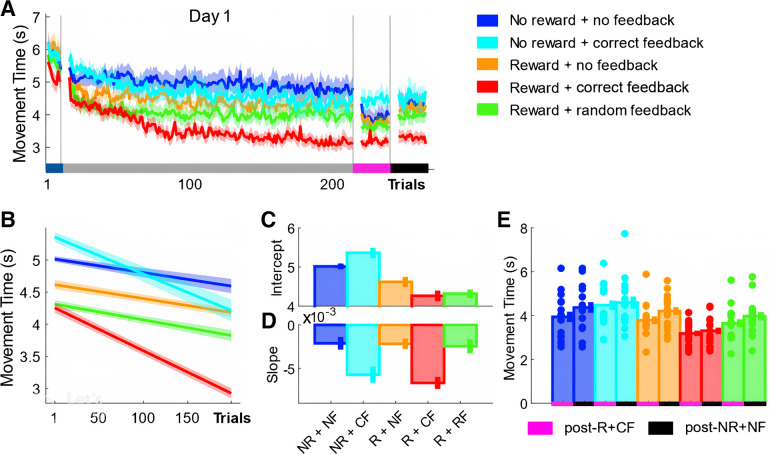
Monetary incentive and performance-based feedback have distinct effects on movement time (MT). *A*: trial-by-trial changes in MT averaged over participants for all groups. *B–D*: averaged predicted model fits (simple polynomial model) to bootstrap estimates for each group (*B*) including intercept (*C*) and slope (*D*) values (error bars represent 95% confidence intervals). *E*: postassessment performance (post-R+CF vs. post-NR+NF). Shaded regions/error bars represent SE. CF, correct performance-based feedback; NF, no performance-based feedback; NR, no monetary incentive; R, monetary incentive; RF, random performance-based feedback.

MT reflected total movement duration from exiting the start box until reaching the last target. We found no difference at baseline (ANOVA; group, *F* = 1.35, *P* = 0.2603). To assess whether monetary incentive and performance-based feedback have distinct effects on MT performance during training, we computed 1,000 bootstrap estimates of the data for each group. Each estimate represented a randomly generated data set (*N* = 15) with replacement. Three separate models were fit to the mean trial-by-trial training data (200 trials) for each bootstrap estimate: *1*) a simple polynomial model [*f*(*x*) = *p1* × *x* + *p2*]; *2*) an exponential model [*f*(*x*) = *a* · exp(*b* · *x*)], and *3*) a power model [*f*(*x*) = *a* × *x*^*b*]. Goodness of fit (*R*^2^) values were extracted for each model fit and were subsequently averaged across groups to determine which model best represented the data. We used 95% confidence intervals (CIs) to test for significant differences between the models. The goodness of fit results showed that there were no statistical differences between models. However, a polynomial model explained the data marginally better than the exponential or power model (Poly1,1 *R*^2^ = 0.6257, CI = [0.5489 0.7025]; Exp1, *R*^2^ = 0.6055, CI = [0.5253 0.6857]; Power1, R^2^ = 0.5852, CI = [0.5137 0.6567]). Therefore, a polynomial model was used in all subsequent analysis, with the two model parameters (*p1*: gradient and *p2*: intercept) being used to assess group differences at the beginning of (intercept) and across training (slope) (see methods) (39). Our results highlight that monetary incentive rapidly enhanced sequential reaching behavior (Fig. 2, *A* and *B*). Specifically, groups who received monetary incentive (Group_-R+CF_, MT = 4.3262, CI = [4.2162 4.4362]; Group_-R+NF_, MT = 4.6860, CI = [4.5616 4.8104]; Group_-R+RF_, MT = 4.3660, CI = [4.2567 4.4757]) exhibited lower MTs at the start of training than the NR groups (Group_-NR+NF_, MT = 5.138, CI = [5.0191 5.2570]; Group_-NR+CF_, MT = 5.3346, CI = [5.2389 5.4303]; intercept; Fig. 2, *B* and *C*). We ran an additional analysis to ascertain that the intercept results indeed reflect a rapid MT improvement: we calculated the mean MT values for each participant only looking at the first 5 trials (which we consider to be short enough to detect rapid changes in performance). A between-groups ANOVA with two grouping factors, *1*) Reward versus No Reward and *2*) Feedback versus No Feedback, revealed a significant effect only for Reward (*F* = 8.2, *P* = 0.0055) and not for Feedback (*F* = 0.05, *P* = 0.8319). These additional analyses align with our previous results and confirm that the intercept results are meaningful and not an artifact of the model fit.

In contrast, only groups who received accurate performance-based feedback (Group_-R+CF_, MT = −0.0072, CI = [−0.0078 −0.0066]; Group_-NR+CF_, MT = −0.0059, CI = [−0.0066 −0.0051]) showed greater learning-related decreases in MT across training, which suggests that feedback has to match performance to enhance learning (Group_-R+RF_, MT = −0.0028, CI = [−0.0034 −0.0022]; slope; Fig. 2, *B* and *D*). Importantly, the combination of monetary incentive and accurate performance-based feedback maximized MT improvements (Group_-R+CF_; Fig. 2, *A* and *B*). Note here that these improvements were not related to higher error rates, which were of equal magnitude across groups and were consistently below an average of one error per trial across a group (Supplemental Fig. S1). Additionally, we ran a separate analysis to further ascertain that our results were not driven by performance differences already present at baseline, considering that a nonsignificant baseline ANOVA does not necessarily imply equal performance across groups. To this end, we baseline corrected the data on an individual basis and ran our analysis again. The results highlight that the results presented were not driven by systemic baseline differences. In line with the original results, we found that groups who received monetary incentive (Group_-R+CF_, MT = −1.0220, CI = [−1.0857 −0.9583]; Group_-R+NF_, MT = −1.4023, CI = [−1.4848 −1.3197]; Group_-R+RF_, MT = −1.6797, CI = [−1.8583 −1.5011]) exhibited lower MTs at the start of training compared with the NR groups (Group_-NR+NF_, MT = −0.6498, CI = [−0.7635 −0.5360]; Group_-NR+CF_, MT = −0.6681, CI = [−0.7583 −0.5780]; intercept). Similarly, only groups who received accurate performance-based feedback (Group_-R+CF_, MT = −0.0061, CI = [−0.0065 −0.0057]; Group_-NR+CF_, MT = −0.0050, CI = [−0.0056 −0.0043]; slope) showed greater learning-related decreases in MT across training (Group_-NR+NF_, MT = −0.0022, CI = [−0.0026 −0.0017]; Group_-R+NF_, MT = −0.0024, CI = [−0.0031 −0.0018]; Group_-R+RF_, MT = −0.0018, CI = [−0.0023 −0.0013]). Yet again, the combination of monetary incentive and accurate performance-based feedback maximized behavioral gains (Group_-R+CF_). Furthermore, we checked whether the reported model results were solely driven by the first training trials by rerunning the same analysis for MT without including the first 5 trials. The results highlight that the model fit is robust and is not driven by the first few (1:5) trials. In line with the original results, we found that groups who received monetary incentive (Group_-R+CF_, MT = 4.1275, CI = [4.0283 4.2267]; Group_-R+NF_, MT = 4.6330, CI = [4.5277 4.7383]; Group_-R+RF_, MT = 4.1830, CI = [4.0583 4.3077]) exhibited lower MTs compared with the NR groups (Group_-NR+NF_, MT = 5.1882, CI = [5.0516 5.3248]; Group_-NR+CF_, MT = 5.1480, CI = [5.0215 5.3248]). Similarly, only groups who received accurate performance-based feedback (Group_-R+CF_, MT = −0.0059, CI = [−0.0062 −0.0055]; Group_-NR+CF_, MT = −0.0045, CI = [−0.0051 −0.0039]) showed greater learning-related decreases in MT across training compared with the other groups (Group_-NR+NF_, MT = −0.0022, CI = [−0.0026 −0.0018]; Group_-R+NF_, MT = −0.0022, CI = [−0.0028 −0.0016]; Group_-R+RF_, MT = −0.0012, CI = [−0.0016 −0.007]). Yet again, the combination of monetary incentive and accurate performance-based feedback maximized behavioral gains (Group_-R+CF_). Importantly, a significant main effect for both time point [Baseline (all trials) vs. post-NR+NF (all trials); mixed-effect ANOVA; *F* = 165.48, *P* < 0.0001] and group (*F* = 3.13, *P* = 0.0199) was found. This suggests that the MT improvements across training reflect true learning, which is still apparent during post-NR+NF.

Across postassessments, we found a significant main effect for both time point [mixed-effect ANOVA; time point post-R (all 20 trials) vs. post-NR+NF (all 20 trials), *F* = 26.32, *P* < 0.0001; Fig. 2*E*) and group (group, *F* = 4.56, *P* = 0.0025). Specifically, post hoc analysis revealed that Group_-R+CF_ was faster than both NR groups (Wilcoxon test; Group_-R+CF_ vs. Group_-NR+NF_, *Z* = −1.3, *P* = 0.0011; Group_-R+CF_ vs. Group_-NR+CF_, *Z* = 0.9, *P* = 0.0358). However, no further post hoc group comparisons yielded any significant results.

### Combined Changes in Maximum and Minimum Velocity Mediate Improvements in MT

**Figure 3.**
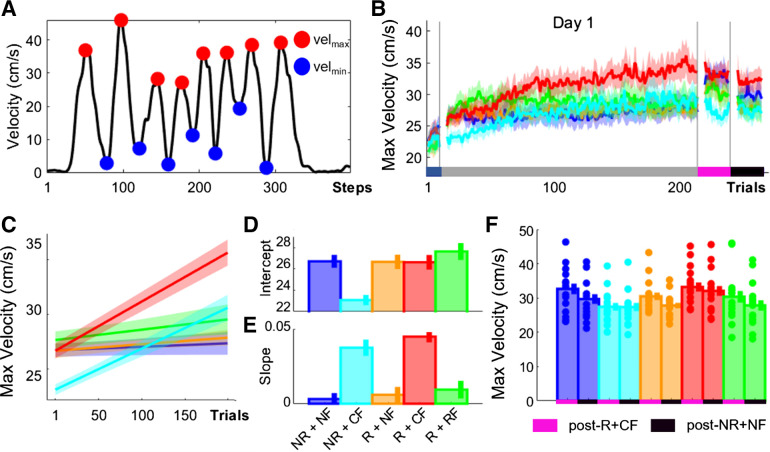
Accurate performance-based feedback led to training-dependent improvements in maximum velocity (vel_max_). *A*: illustration of vel_max_ and minimum velocity (vel_min_). *B*: trial-by-trial changes in vel_max_ averaged over participants for all groups. *C–E*: averaged predicted model fits (simple polynomial model) to bootstrap estimates for each group (*C*) including intercept (*D*) and slope (*E*) values (error bars represent 95% confidence intervals). *F*: postassessment performance (post-R+CF vs. post-NR+NF). Shaded regions/error bars represent SE. CF, correct performance-based feedback; NF, no performance-based feedback; NR, no monetary incentive; R, monetary incentive; RF, random performance-based feedback.

It has been shown that improvements in MT can be achieved via increases in maximum velocity (vel_max_) during simple discrete reaching movements (3, 4). To assess the effects of monetary incentive and performance-based feedback on vel_max_ (see methods, *Eq. 1*), we averaged vel_max_ across the eight reaching movements (Fig. 3*A*). We found no difference at baseline (ANOVA; group, *F* = 0.76, *P* = 0.5532) and an unclear result during early training (intercept; Fig. 3, *C* and *D*).

At the beginning of training, Group_-NR+CF_ scored lowest on vel_max_ (Group_-NR+CF_, vel_max_ = 23.3461, CI = [22.8807 23.8115]), whereas Group_-NR+NF_ scored vel_max_ values similar to the other groups (Group_-NR+NF_, vel_max_ = 26.4338, CI = [25.8331 27.0345]; Group_-R+CF_, vel_max_ = 26.8560, CI = [26.1144 27.5976]; Group_-R+NF_, vel_max_ = 26.8644, CI = [25.2824 27.4464]; Group_-R+RF_, vel_max_ = 27.7592, CI = [26.9189 28.5994]). In contrast, accurate performance feedback was associated with a pronounced learning-related increase in vel_max_ (i.e., slope; Group_-R+CF_, vel_max_ = 0.0438, CI = [0.0402 0.0474]; Group_-NR+CF_, vel_max_ = 0.0354, CI = [0.0305 0.0404]; slope; Fig. 3, *C* and *E*) compared with the other groups (Group_-NR+NF_, vel_max_ = 0.0022, CI = [0.0002 0.0043]; Group_-R+NF_, vel_max_ = 0.0044, CI = [0.0005 0.0083]; Group_-R+RF_, vel_max_ = 0.0089, CI = [0.0065 0.0113]). Importantly, and similarly to MT, the combination of monetary incentive and accurate performance-based feedback maximized the gains observed in vel_max_ (Group_-R+CF_; Fig. 2, *B* and *C*). Additionally, a significant main effect for time point was found when comparing performance between Baseline and post-NR+NF (mixed-effect ANOVA; *F* = 88.92, *P* < 0.0001) but not for group (*F* = 1.47, *P* = 0.2201). This highlights again that peak velocity (vel_peak_) improvements across training may reflect true learning. Across postassessments, we found a significant main effect for time point (mixed-effect ANOVA; time point post-R vs. post-NR+NF, *F* = 31.25, *P* < 0.0001; Fig. 2*E*) but not for group (*F* = 1.84, *P* = 0.1304).

**Figure 4.**
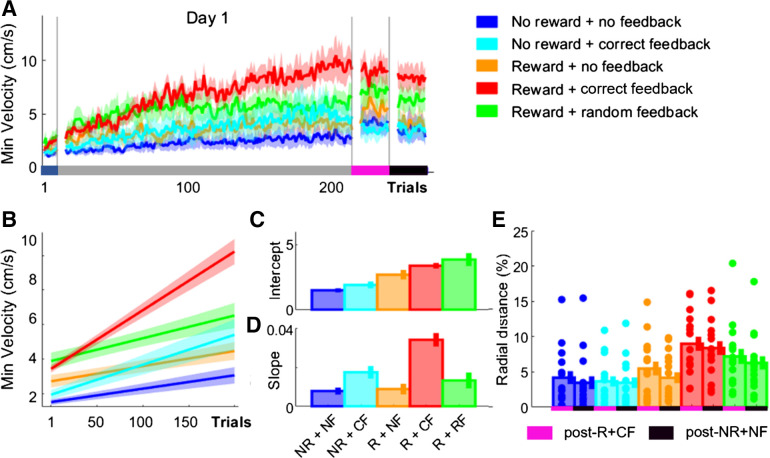
Accurate performance feedback led to training-dependent improvements in minimum velocity (vel_min_). *A*: trial-by-trial changes in vel_min_ averaged over participants for all groups. *B–D*: averaged predicted model fits (simple polynomial model) to bootstrap estimates for each group (*B*) including intercept (*C*) and slope (*D*) values (error bars represent 95% confidence intervals). *E*: postassessment performance (post-R+CF vs. post-NR+NF). Shaded regions/error bars represent SE. CF, correct performance-based feedback; NF, no performance-based feedback; NR, no monetary incentive; R, monetary incentive; RF, random performance-based feedback.

In contrast to discrete motor behaviors, the task utilized for this study consisted of a sequence of reaching movements. Therefore, improvements in MT could additionally be driven by a reduction in dwell times when transitioning between reaching movements. To assess the effect of monetary incentive and performance-based feedback on dwell times, we obtained minimum velocity (vel_min_) values and averaged them across the 7 reaching transitions (see methods, *Eq. 2*; Fig. 3*A*). Whereas no differences at Baseline were observed (ANOVA; group, *F* = 1.26, *P* = 0.2923), monetary incentive enhanced vel_min_ during early training (intercept; Group_-R+CF_, vel_min_ = 3.6630, CI = [3.4047 3.9212]; Group_-R+NF_, vel_min_ = 2.6834, CI = [2.3229 3.0439]; Group_-R+RF_, vel_min_ = 3.9792, CI = [3.5149 4.4436]; intercept; Fig. 4, *B* and *C*). In contrast to vel_max_, Group_-NR+CF_ showed higher vel_min_ values than Group_-NR+NF_, which were close to Group_-R+NF_ (intercept; Group_-NR+CF_, vel_min_ = 2.1124, CI = [1.7984 2.4263]; Group_-NR+NF_, vel_min_ = 1.5604, CI = [1.3579 1.7630]). Additionally, performance feedback was associated with a learning-related increase in vel_min_ (slope; Group_-R+CF_, vel_min_ = 0.0322, CI = [0.0290 0.0355]; Group_-NR+CF_, vel_min_ = 0.0160, CI = [0.0130 0.0190]; slope; Fig. 4, *B* and *D*) compared with no feedback (Group_-NR+NF_, vel_min_ = 0.0072, CI = [0.0057 0.0087]; Group_-R+NF_, vel_min_ = 0.0083, CI = [0.0126 0.0188]). Interestingly, random feedback in Group_-NR+RF_ led to similar learning slopes compared with Group_-NR+CF_ (Group_-R+RF_, vel_min_ = 0.0126, CI = [0.0088 0.0165]), which may suggest that improvements in vel_min_ require feedback irrespective of whether it is accurate. Importantly, similarly to both MT and vel_max_, combining monetary incentive with accurate performance feedback maximized the gains observed in vel_min_ (Group_-R+CF_; Fig. 3, *A* and *C*). Additionally, a significant main effect for time point was found when comparing performance between Baseline and post-NR+NF (mixed-effect ANOVA; *F* = 72.33, *P* < 0.0001) and for group (*F* = 4.14, *P* = 0.0046), with a significant interaction between them (*F* = 4.01, *P* = 0.0055). A post hoc analysis comparing changes in performance from Baseline to post-NR+NF revealed that Group_-R+CF_ was faster than both NR groups (Wilcoxon test; Group_-R+CF_ vs. Group_-NR+NF_, *Z* = −3.071, *P* = 0.0121; Group_-R+CF_ vs. Group_-NR+CF_, *Z* = 3.033, *P* = 0.0121) and Group_-R+NF_ (Group_-R+CF_ vs. Group_-R+NF_, *Z* = −2.612, *P* = 0.0299). However, no further post hoc group comparisons yielded any significant results. These results highlight that vel_min_ improvements across training may reflect true learning, which is still apparent during post-NR+NF.

Across postassessments, we found a significant main effect for both time point (mixed-effect ANOVA; time point post-R vs. post-NR+NF, *F* = 19.04, *P* < 0.0001; Fig. 4*E*) and group (group, *F* = 4.41, *P* = 0.0003). Specifically, post hoc analysis revealed that Group_-R+CF_ had higher vel_min_ than both NR groups (Wilcoxon test; Group_-R+CF_ vs. Group_-NR+NF_, *Z* = −0.81, *P* = 0.0108; Group_-R+CF_ vs. Group_-NR+CF_, *Z* = 1.01, *P* = 0.0075). However, no further significant differences between groups were found. These results suggest that monetary incentive and performance-based feedback have distinct effects on performance during a complex, sequential reaching task. Monetary incentive led to a rapid decrease in MTs, whereas performance feedback was associated with a learning-dependent decrease in MT. This pattern was also observed in vel_max_ and vel_min_, which suggests that the combined changes in both underlie the MT results. Crucially, combining monetary incentive with accurate performance feedback maximized the behavioral gains observed.

### Movement Fusion Is Associated with Additional Performance Gains in MT

**Figure 5.**
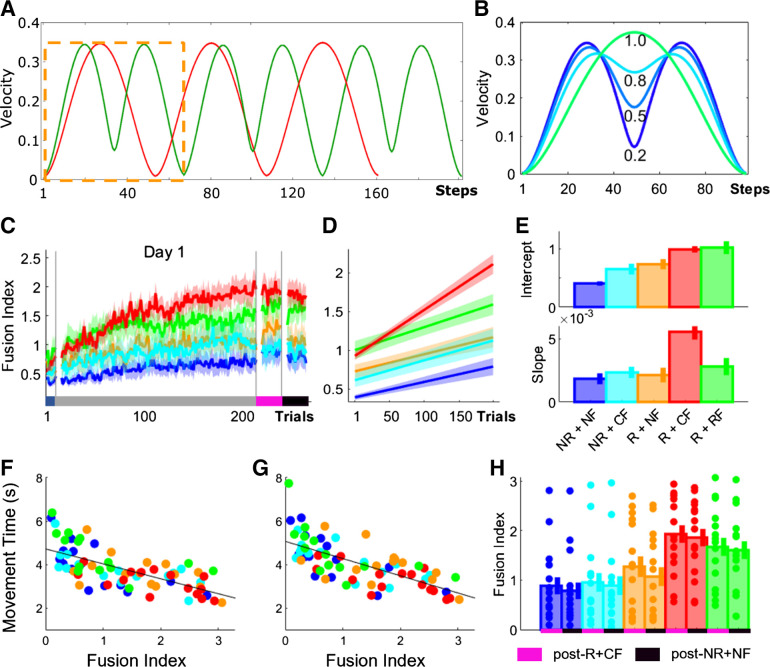
Movement fusion is strongly associated with improvements in movement time (MT). *A*: illustration of a velocity profile corresponding to executing 6 reaching movements individually (green) and 3 fused reaches (red). Stop period, apparent when executing individual movements, disappears when executing a fully fused reaching movement (dashed orange). *B*: illustration of fusion index (FI). *C*: trial-by-trial changes in FI averaged over participants for all groups. *D* and *E*: averaged predicted model fits (simple polynomial model) to bootstrap estimates for each group (*D*) including intercept and slope values (*E*) (error bars represent 95% confidence intervals). *F* and *G*: scatterplots displaying the relationship between MT and FI levels during post-R+CF (*F*) and post-NR+NF (*G*) with a linear line fitted across groups. *H*: postassessment performance (post-R+CF vs. post-NR+NF). Shaded regions/error bars represent SE. CF, correct performance-based feedback; NF, no performance-based feedback; NR, no monetary incentive; R, monetary incentive; RF, random performance-based feedback.

Thus, combining monetary incentive with accurate performance-based feedback led to faster MTs by increasing both vel_max_ and vel_min_. One strategy to achieve faster reaching movements while simultaneously reducing dwell times when transitioning between reaches is movement fusion. Fusion describes the blending of individual motor elements into a combined action (26–28). This is represented in the velocity profile by the stop period between two movements gradually disappearing and being replaced by a single velocity peak (Fig. 5*A*). To measure movement fusion, we developed a fusion index (FI; methods, *Eq. 3*) that compared the mean vel_max_ of two sequential reaches with the vel_min_ around the via point (transition). The smaller the difference between these values, the greater fusion had occurred of these two movements, as reflected by a FI value closer to 1 (Fig. 5*B*).

Considering that participants completed 7 transitions to complete a trial, the maximum FI value was 7. We found no difference at Baseline (ANOVA; group, *F* = 1.36, *P* = 0.2555, Fig. 5*C*). Monetary incentive in combination with performance-based feedback (both correct and random) enhanced movement fusion during early training (intercept; Group_-R+CF_, FI = 0.9855, CI = [0.9280 1.0429]; Group_-R+RF_, FI = 1.0160, CI = [0.9075 1.1245]; intercept; Fig. 5, *D* and *E*). In contrast, intercept differences between Group_-R+NF_ and Group_-NR+CF_ were considerably smaller and closer to Group_-NR+NF_ (Group_-R+NF_, FI = 0.7225, CI = [0.6309 0.8141]; Group_-NR+CF_, FI = 0.6419, CI = [0.5498 0.7341]; Group_-NR+NF_, FI = 0.4034, CI = [0.3650 0.4419]). This suggests that early increases in movement fusion may depend on the availability of performance correct feedback in combination with reward. Importantly, in comparison to all other groups, only Group_-R+CF_ exhibited a pronounced learning-related increase in FI that was greater than in Group_-NR+CF_ and Group_-R+RF_ (Group_-R+CF_, FI = 0.0056, CI = [0.0050 0.0062]; Group_-NR+CF_, FI = 0.0022, CI = [0.0016 0.0028]; Group_-R+RF_, FI = 0.0028, CI = [0.0021 0.0035]). Furthermore, Group_-NR+CF_ and Group_-R+RF_ were not different from the NR groups (Group_-NR+CF_, FI = 0.0024, CI = [0.0019 0.0028]; Group_-NR+NF_, FI = 0.0018, CI = [0.0014 0.0022]; slope; Fig. 5, *D* and *E*). Additionally, a significant main effect for time point was found when comparing performance between Baseline and post-NR+NF (mixed-effect ANOVA; *F* = 75.68, *P* < 0.0001) and for group (*F* = 3.9426, *P* = 0.2201), with a significant interaction between them (*F* = 4.38, *P* = 0.0032). A post hoc analysis comparing changes in performance from Baseline to post-NR+NF revealed that Group_-R+CF_ exhibited higher FI values than both NR groups (Wilcoxon test; Group_-R+CF_ vs. Group_-NR+NF_, *Z* = −2.986, *P* = 0.0141; Group_-R+CF_ vs. Group_-NR+CF_, *Z* = 3.121, *P* = 0.0141). However, no further post hoc group comparisons yielded any significant results. These results highlight that FI improvements across training may reflect true learning, which is still apparent during post-NR+NF. Importantly, when correlating FI and MT during both post-R+CF (Fig. 5*F*) and post-NR+NF (Fig. 5*G*) we found that higher FI values were associated with MT while accounting for the factor group (post-R+CF: ρ = −0.702, *P* < 0.0001; post-NR+NF: ρ = −0.694, *P* < 0.0001). This highlights that faster MTs are related to increased fusion.

Across postassessments we found a significant main effect for both time point (mixed-effect ANOVA; time point post-R+CF vs. post-NR+NF, *F* = 12.48, *P* < 0.0001; Fig. 5*H*) and group (group, *F* = 4.85, *P* = 0.0017). Specifically, post hoc analysis revealed that Group-R+CF had higher FI values than both NR groups (Wilcoxon test; Group-R+CF vs. Group-NR+NF, *Z* = −0.70, *P* = 0.0146; Group_-R+CF_ vs. Group_-NR+CF_, *Z* = 1.06, *P* = 0.0050). However, no further significant differences were found.

### Monetary Incentive in Combination with Performance-Based Feedback Led to Significant Improvements in Performance across Multiple Days

To investigate whether these findings could be replicated and to further assess the kinematic underpinnings of movement fusion, we conducted a second experiment using the same task design. In *experiment 2*, only Group_-R+CF_ and Group_-NR+NF_ were included, to contrast the most beneficial feedback regime (i.e., monetary incentive with accurate performance feedback) with its logical opposite. Additionally, we added a further testing day (*day 2*) to investigate whether movement fusion can be further enhanced with additional training. During *day 2*, participants underwent the same experimental protocol as *day 1* (Fig. 1*C*). We did not observe any differences during Baseline for any measure (MT, Wilcoxon test, Z = −1.38, *P* = 0.17, Supplemental Fig. S2a; vel_max_, Wilcoxon test, *Z* = 0.70, *P* = 0.4812, Supplemental Fig. S2b; vel_min_, Wilcoxon test, *Z* = 1.44, *P* = 0.1516, Supplemental Fig. S2c; FI, Wilcoxon test, *Z* = 1.31, *P* = 0.1908, Supplemental Fig. S2d).

**Figure 6.**
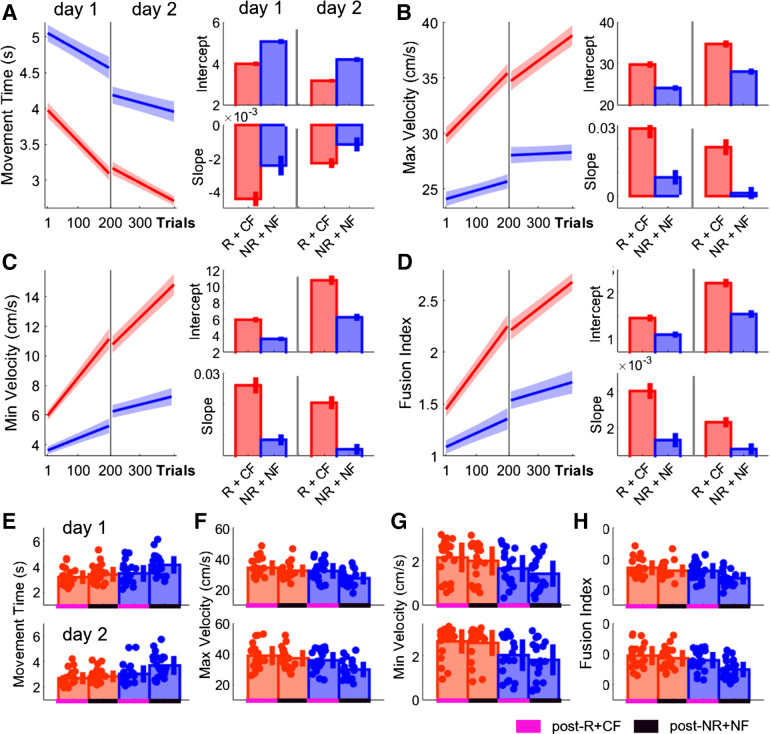
Monetary incentive and correct performance feedback invigorate initial performance and enhances learning across both testing days. *A–D*: averaged predicted model fits (simple polynomial model) to bootstrap estimates for each group including intercept and slope values (error bars represent 95% confidence intervals) for movement time (MT; *A*) maximum velocity (vel_max_; *B*) minimum velocity (vel_min_; *C*) and fusion index (FI; *D*). *E–H*: postassessment performance comparing group performance during post-R+CF and post-NR+NF on *day 1* (*top*) and *day 2* (*bottom*) for MT (*E*), vel_max_ (*F*), vel_min_ (*G*), and FI (*H*) (shaded regions/error bars represent SE). CF, correct performance-based feedback; NF, no performance-based feedback; NR, no monetary incentive; R, monetary incentive; RF, random performance-based feedback.

Instead, we found that monetary incentive in combination with performance-based feedback enhanced MT performance during early training on *day 1* (Group_-R+CF_, MT = 3.8663, CI = [3.7725 3.9602]; Group_-NR+NF_, MT = 4.9663, CI = [4.8428 5.0898]; intercept; Fig. 6*A*). Similarly, Group_-R+CF_ exhibited higher intercepts for vel_max_ and vel_min_ (Group_-R+CF_, vel_max_ = 30.4803, CI = [29.6730 31.2876], Group_-NR+NF_, vel_max_ = 24.5439, CI = [23.8895 25.1983], intercept, Fig. 6*B*; Group_-R+CF_, vel_min_ = 6.4902, CI = [6.1319 6.8486], Group_-NR+NF_, vel_min_ = 3.7302, CI = [3.4472 4.0132], intercept, Fig. 6*C*). Additionally, we found that Group_-R+CF_ also showed higher levels of movement fusion (Group_-R+CF_, FI = 1.5378, CI = [1.4604 1.6151], Group_-NR+NF_, FI = 1.0930, CI = [1.0260 1.1599]; intercept; Fig. 6*D*). Importantly, we observed that Group_-R+CF_ exhibited steeper learning curves across all measures on *day 1* (Group_-R+CF_, MT = −0.0036, CI = [−0.0040 −0.0032], Group_-NR+NF_, MT = −0.0018, CI = [−0.0023 −0.0012], slope, Fig. 6*A*; Group_-R+CF_, vel_max_ = 0.0230, CI = [0.0192 0.0268], Group_-NR+NF_, vel_max_ = 0.0045, CI = [0.0017 0.0074], slope, Fig. 6*B*; Group_-R+CF_, vel_min_ = 0.0217, CI = [0.0192 0.0242], Group_-NR+NF_, vel_min_ = 0.0075, CI = [0.0058 0.0092], slope, Fig. 6*C*; (Group_-R+CF_, FI = 0.0034, CI = [0.0029 0.0038], Group_-NR+NF_, FI = 0.0013, CI = [0.0009 0.0016], slope, Fig. 6*D*). These results replicate our findings from *experiment 1* and highlight that monetary incentive in combination with performance-based feedback both invigorates performances during early training and enhances learning leading to additional performance gains across training. Furthermore, results from *experiment 2* showed that performance can be further improved across an additional testing day only if monetary incentive in combination with performance-based feedback was provided (Group_-R+CF_, MT = −0.0020, CI = [−0.0022 −0.0017], Group_-NR+NF_, MT = −0.0011, CI = [−0.0015 −0.0007], slope, Fig. 6*A*; Group_-R+CF_, vel_max_ = 0.0181, CI = [0.0150 0.0211], Group_-NR+NF_, vel_max_ = 0.0016, CI = [−0.0008 0.0041], slope, Fig. 6*B*; Group_-R+CF_, vel_min_ = 0.0173, CI = [0.0153 0.0192], Group_-NR+NF_, vel_min_ = 0.0046, CI = [0.0030 0.0062], slope, Fig. 6*C*; Group_-R+CF_, FI = 0.0020, CI = [0.0017 0.0022], Group_-NR+NF_, FI = 0.0008, CI = [0.0005 0.0011], slope, Fig. 6*D*).

Across postassessments, we found significant interactions between time point (post-R+CF vs. post-NR+NF) and group (Group_-R+CF_ vs. Group_-NR+NF_) on both days for MT (mixed ANOVA; *day 1*: *F* = 18.07, *P* < 0.0001; *day 2*: *F* = 19.99, *P* < 0.0001) and vel_max_ (mixed ANOVA; *day 1*: *F* = 8.02, *P* = 0.0072; *day 2*: *F* = 24.92, *P* < 0.0001). Specifically, there was a significant group difference during post-NR+NF for both MT (Wilcoxon test; *day 1*: *Z* = −2.82, *P* = 0.0192; *day 2*: Z = −3.27, *P* = 0.0044; Fig. 6*E*) and vel_max_ (Wilcoxon test; *day 1*: *Z* = 2.84, *P* = 0.018; *day 2*: *Z* = 3.07, *P* = 0.0084; Fig. 6*F*). However, during post-R+CF no differences were found (MT, Wilcoxon test, *day 1*: *Z* = −1.13, *P* = 1; *day 2*: *Z* = −1.38, *P* = 1; vel_max_, Wilcoxon test, *day 1*: *Z* = 0.86, *P* = 1; *day 2*: *Z* = 0.93, *P* = 1). This indicates that Group_-NR+NF_ were able to rapidly invigorate their performance during post-R+CF. However, these performance gains were not maintained during post-NR+NF, suggesting that they remained transient in nature. Additionally, we found a significant effect for group on both testing days for vel_min_ (mixed ANOVA; *day 1*: *F* = 4.90, *P* = 0.0327; *day 2*: *F* = 7.70, *P* = 0.0083; Fig. 6*G*) and FI (mixed ANOVA; *day 1*: *F* = 4.91, *P* = 0.0324; *day 2*: *F* = 7.38, *P* = 0.0097; Fig. 6*H*). This suggests that the improvements in vel_min_ and FI within Group_-R+CF_ were more stable across postassessments (i.e., when reward was no longer available).

### Spatial Reorganization Identifies the Final Stages of Movement Fusion and Can Be Enhanced through Monetary Incentive in Combination with Performance-Based Feedback

**Figure 7.**
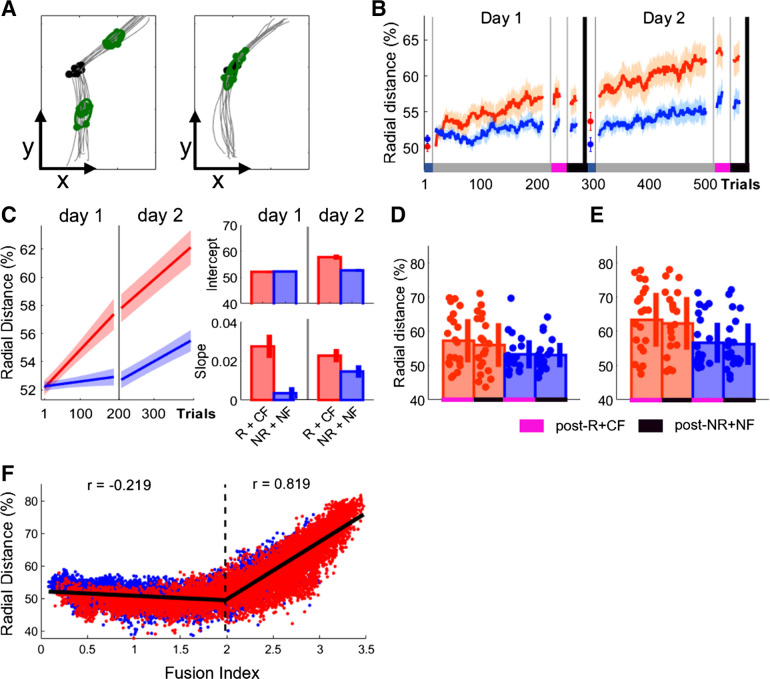
Reward-based improvements in spatial reorganization. *A*: example data for the spatial location of maximum velocity (vel_max_) when performing 2 individual (*left*) and 1 fused (*right*) reaching movement. *B*: trial-by-trial changes in radial distance averaged over participants for both groups. *C*: averaged predicted model fits (simple polynomial model) to bootstrap estimates for each group including intercept and slope values (error bars represent 95% confidence intervals). *D* and *E*: postassessment comparing group performance during post-R+CF and post-NR+NF on *day 1* (*D*) and *day 2* (*E*) (shaded regions/error bars represent SE). *F*: scatterplot illustrating the relationship between mean fusion index (FI) levels and spatial reorganization (radial distance %). It includes a 2-segment piecewise linear function fitted to the data. CF, correct performance-based feedback; NF, no performance-based feedback; NR, no monetary incentive; R, monetary incentive; RF, random performance-based feedback.

The results from both experiments suggest that movement fusion represents a viable strategy to further enhance performance [via increases in reaching speed (vel_max_) and decreases in dwell time between reaches (vel_min_)]. Importantly, movement fusion is a training-dependent process that can be accelerated by providing both monetary incentive and performance-based feedback. Additionally, our findings indicate that fusion is not only associated with performance gains (i.e., faster MTs) during training but also during periods without feedback (post-NR+NF). This may indicate that movement fusion allows for improved retention. To assess whether movement fusion led to a change in how the action is performed spatially, we assessed the radial distance (RD) between vel_max_ on the submovement and vel_min_ around the via points (Fig. 7*A*). When executing a fluid point-to-point reaching movement (i.e., stopping in the target), vel_max_ will be spatially located approximately halfway through the reaching movement (Fig. 7*A*, *right*). However, with movement fusion vel_max_ will drift closer to vel_min_, which is located close to the target (Fig. 7*A*, *left*). Therefore, RD between the two becomes smaller with increasing FI levels, which can be expressed as a percentage of distance covered (Supplemental Fig. S3). Hence, higher RD values represent the two movements merging together. We found that across training Group_-R+CF_ expressed spatial reorganization as seen in a significant decrease in RD between vel_max_ and vel_min_ (Fig. 7*B*).

No difference between groups in RD was observed during Baseline (Wilcoxon test; *Z* = −1.18, *P* = 0.2371; Fig. 7*B*). Instead, we found that monetary incentive in combination with performance-based feedback enhanced performance during early training only on *day 2* (*day 1*: Group_-R+CF_, RD = 52.0447, CI = [51.5142 52.5752], Group_-NR+NF_, RD = 52.2428, CI = [51.9106 52.5751]; *day 2*: Group_-R+CF_, RD = 57.8621, CI = [56.8473 58.8768], Group_-NR+NF_, RD = 52.7398, CI = [52.1450 53.3346]; intercept; Fig. 7*C*). This suggests that spatial reorganization is a learning-dependent process that cannot be rapidly enhanced. Importantly, we found that Group_-R+CF_ exhibited a pronounced learning-related increase in RD across both testing days (*day 1*: Group_-R+CF_, RD = 0.0277, CI = [0.0215 0.338], Group_-NR+NF_, RD = 0.0036, CI = [0.0005 0.0067]; *day 2*: Group_-R+CF_, RD = 0.0232, CI = [0.0198 0.0267], Group_-NR+NF_, RD = 0.0144, CI = [0.0112 0.0175]; slope; Fig. 7*C*). Across postassessments, we found a significant main effect for group on *day 2* (mixed ANOVA; *day 1*: *F* = 3.08, *P* = 0.0868; *day 2*: *F* = 5.76, *P* = 0.0211; Fig. 7, *D* and *E*). However, no main effect for time point was found (*day 1*: *F* = 1.87, *P* = 0.1796; *day 2*: *F* = 2.85, *P* = 0.0989). This suggest that changes in RDs were stable and more pronounced in Group_-R+CF_. To understand the relationship between FI and spatial reorganization, we plotted them against each other and detected a pronounced drift in RD (%) with increasing FI levels resulting in a curvilinear shape (Fig. 7*F*). After fitting a two-segment piecewise linear function to the data, we found an inflection point at ∼1.66 (FI) and a strong correlation between FI and RD for the second segment (partial correlation controlling for group; *segment 1*: ρ = −0.16; *P* < 0.0001; *segment 2*: ρ = 0.89; *P* < 0.0001). This suggests that to fully fuse two consecutive movements spatial reorganization is required (26–28) and this process can be enhanced with a combination of monetary incentive and correct performance-based feedback.

### Movement Fusion Is Associated with Improvements in Smoothness

**Figure 8.**
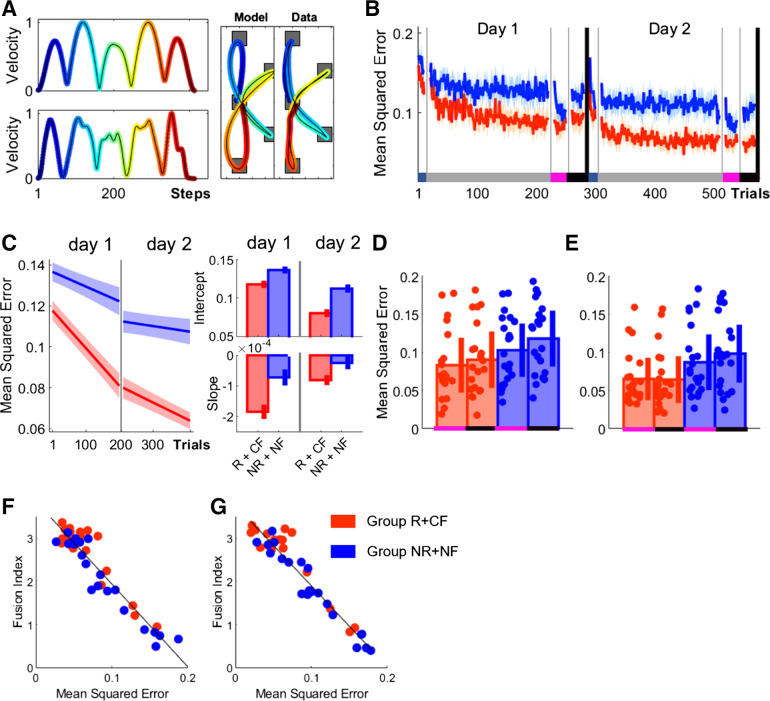
Movement fusion is associated with improvements in movement smoothness. *A*: comparisons between data and the predictions of a minimum-jerk model for both trajectory (*right*) and velocity (*left*) profiles for a single trial. *B*: trial-by-trial changes in mean squared error (MSE) averaged over participants for both groups. *C*: averaged predicted model fits (simple polynomial model) to bootstrap estimates for each group including intercept and slope values (error bars represent 95% confidence intervals). *D* and *E*: postassessment comparing group performance during post-R+CF and post-NR+NF on day 1 (*D*) and day 2 (*E*) (shaded regions/error bars represent SE). *F* and *G*: scatterplot illustrating the relationship between mean fusion index (FI) levels and MSE for post-R+CF (*F*) and post-NR+NF (*G*) (pooled across days). CF, correct performance-based feedback; NF, no performance-based feedback; NR, no monetary incentive; R, monetary incentive; RF, random performance-based feedback.

As movement fusion involves the difference between vel_max_ and vel_min_ decreasing (Fig. 5*B*), it implies that periods of acceleration/deceleration should become less pronounced and the movement ought to become smoother. To assess whether movement fusion is associated with increases in smoothness, participants’ performance was compared to the predictions of an optimization model that minimized jerk across the movement sequence (30). On trial-by-trial basis, mean squared error (MSE) was calculated between the model and the actual velocity profile (methods, *Eq. 4*; Fig. 8*A*). In summary, Group_-NR+NF_ became more aligned to the model’s predictions, suggesting that this group’s performance became smoother (Fig. 8*B*).

No difference between groups in MSE was observed during Baseline (Wilcoxon test; *Z* = −1.16, *P* = 0.2472). Instead, we found that monetary incentive in combination with performance-based feedback enhanced performance during early training on both days (*day 1*: Group_-R+CF_, MSE = 0.1178, CI = [0.1130 0.1227], Group_-NR+NF_, MSE = 0.1372, CI = [0.1326 0.1417]; *day 2*: Group_-R+CF_, MSE = 0.0796, CI = [0.0743 0.0850], Group_-NR+NF_, MSE = 0.1129, CI = [0.1078 0.1180]; intercept; Fig. 8*C*). These results were in line with the movement fusion results and highlighted that R+CF enhanced movement smoothness during early training. Importantly, we found that Group_-R+CF_ exhibited a pronounced learning-related decrease in MSE across both testing days (*day 1*: Group_-R+CF_, MSE = −0.0018, CI = [−0.0021 −0.0016], Group_-NR+NF_, MSE = −0.0007, CI = [−0.0010 −0.0005]; *day 2*: Group_-R+CF_, MSE = −0.0008, cCI = [−0.0010 −0.0006], Group_-NR+NF_, MSE = −0.0003, CI = [−0.0005 −0.0001]; slope; Fig. 8*C*). Additionally, during both post-R+CF (Fig. 8*F*) and post-NR+NF (Fig. 8*G*) we found that higher FI values are strongly associated with reduced MSE while accounting for the factor group (post-R+CF; ρ = −0.944, *P* < 0.0001; post-NR+NF; ρ = −0.952, *P* < 0.0001).

This suggests that increases in movement fusion were related to improvements in smoothness. Further support comes from analysis that showed that R+CF also reduced spectral arc length, an alternative measure of smoothness [see methods (37, 43)] (Supplemental Fig. S4).

Across postassessments, we found a significant main effect for group on *day 2* (mixed ANOVA; *day 1*: *F* = 3.08, *P* = 0.0868; *day 2*: *F* = 5.76, *P* = 0.0211; Fig. 8, *D* and *E*). However, no main effect for time point was found (*day 1*: *F* = 1.87, *P* = 0.1796; *day 2*: *F* = 2.85, *P* = 0.0989), which suggests that changes in RDs were stable and more pronounced in Group_-R+CF_.

### Performance Gains Are Maintained across an Additional Testing Day without Reward

We next aimed to assess the robustness of these performance gains in an experiment including an additional testing day without any monetary incentive and performance feedback (elongated washout condition; *N* = 5). Participants underwent the same regime as in *experiment 2* and on *day 3* were asked to complete 200 no-reward/feedback trials. Even after 24 h, and over the course of 200 additional NR+NF trials, participants maintained similar MT performance levels.

**Figure 9.**
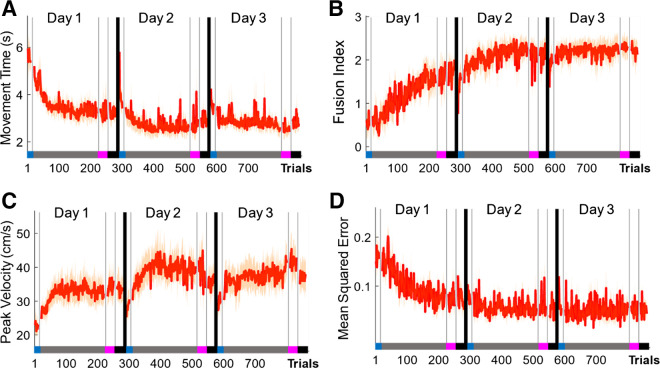
Long-term maintenance of performance without feedback. *A–D*: *experiment 3*. Median data across all 3 days [early (first 20 trials) vs. late (last 20 trials) training]. *A*: movement time (MT). *B*: fusion index (FI) level. *C*: maximum velocity (vel_max_). *D*: mean squared error between data and minimum-jerk model prediction. Shaded regions represent SE.

We used a repeated-measures ANOVA with time point [early (first 15 trials) vs. late (last 15 trials) across all testing days] as the within factor to assess changes across testing days (repeated-measures ANOVA, *F* = 28.65, *P* < 0.0001; Fig. 9*A*). These results indicate that performance improved over the course of the first 2 days (i.e., when reward was provided). However, no changes in MT performance could be observed between late training on *day 2* and early training on *day 3* (Wilcoxon test, *Z* = −1.21, *P* = 0.3016) and between early and late training on *day 3* (Wilcoxon test, *Z* = −1.48, *P* = 0.4444). Similarly, fusion levels appeared stable across the additional testing day without feedback (repeated-measures ANOVA, *F* = 19.19, *P* < 0.0001; Fig. 9*B*), with no changes in performance between late *day 2* and early *day 3* (Wilcoxon test, *Z* = −0.94, *P* = 1) and no changes across *day 3* (Wilcoxon test; early vs. late; *day 3*, Z = −0.67, *P* = 1). In addition, vel_max_ values were maintained transitioning to and across *day 3* (Wilcoxon test, late training *day 2* × early training *day 3*, *Z* = −1.21, *p* = 1; early training *day 3* × late training *day 3*, *Z* = −1.21, *P* = 1; Fig. 9*C*). When assessing changes in smoothness, we found that performance aligned progressively with the predictions of the minimum-jerk model (repeated-measures ANOVA, *F* = 23.38 *P* < 0.0001; Fig. 9*D*), whereas no significant changes in similarity could be observed transitioning to and across *day 3* (Wilcoxon test, late training *day 2* × early training *day 3*, *Z* = −2.02, *P* = 1; early training *day 3* × late training *day 3*, *Z* = −1.21, *P* = 1).

## DISCUSSION

Previous research on the effects of reward on motor behavior found that reward invigorated performance (1, 3–5, 11–14) or enhanced motor learning and/or retention (8–10, 15–17, 44). More specifically, studies using saccadic or discrete reaching movements have consistently shown that reward rapidly improved MTs while maintaining similar levels of accuracy (1, 3–5, 11–14), whereas most research employing force and button-press tasks found reward-related improvements in learning and/or retention (i.e., reductions in error rates or increases in number of successful trials) (8–10, 15–17). Our results suggest that these reward-related effects represent dissociable mechanisms and can be attributed to monetary incentive and performance feedback, respectively. *Experiment 1* showed that monetary incentive rapidly reduced MT during early training, whereas accurate performance feedback led to training-related improvements in MT irrespective of reward availability. Crucially, combining monetary incentive with performance feedback resulted in both a rapid reduction and a learning-related improvement in MT that maximized performance gains.

The rapid effect of monetary incentive on motor behavior has previously been explained by reward paying the energetic cost of enhanced performance (1, 3, 4). For example, faster discrete reaching movements under reward conditions have been associated with increased arm stiffness (4). Although an attractively simple strategy, increased stiffness comes with a marked escalation in metabolic costs (45). Interestingly, these effects of reward are transient in that they are no longer observed once reward is removed (1, 3, 4). Therefore, monetary incentive seems to enable the transient use of energetically demanding [or even cognitively demanding (1, 14)] control mechanisms. Neurally this may relate to prospective reward altering the trial-by-trial excitability of several motor regions (dorsal premotor cortex, primary motor cortex), irrespective of past reward history (46). In humans, it has even been shown that trial-by-trial primary motor cortex excitability reflects the subjective value of reward and also mediates its incentivized effects on motor performance (47)

In contrast, performance-based feedback led to training-dependent improvements in MT. These findings are in line with previous work using force and button-press tasks that showed learning-dependent improvements in performance (8–10, 15–17). Performance feedback, provided by either points or binary feedback, represents a reinforcement-based teaching signal (implicit reward signal) (18, 19, 21) that provides information on how well a motor task has been completed (knowledge of performance) and has been shown to enhance other forms of motor learning (11, 20–24) and retention (9–11). Interestingly, recent research has shown that subpopulations of neurons in the primary motor cortex specialize over training and signal outcome information of a single trial but are independent of reward and movement kinematics (48). This suggests that feedback information is present in motor cortices alongside neurons that encode reward and could indicate that monetary incentive and performance feedback have dissociable impact on the motor system.

Within this context, it is important to highlight that performance improvements do not necessarily have to reflect motor learning but could be driven by transient factors such as arm stiffness, cocontraction, and/or task knowledge. Our retention analysis shows that all groups improved their performance, comparing Baseline and post-NR performance. This indicates that the observed performance improvements may reflect “true” motor learning that is stable across periods without monetary reward and feedback available.

Crucially, monetary incentive in combination with accurate performance feedback resulted in both a rapid reduction and a learning-related improvement in MT that maximized performance gains. These findings are in line with recent research showing that such a combination led to enhanced learning and improvements in retention in a pinch force reproduction task (15). It has been suggested that monetary incentive augments exploitative behavior after successful feedback (i.e., reproduces successful behavior) and increases explorative behavior after unsuccessful feedback (i.e., magnitude of behavioral change) (49). In other words, monetary incentive boosts the reinforcement learning occurring with performance feedback (15, 49). Interestingly, monetary incentive in combination with random performance feedback did not lead to learning-related improvements. Within the context of reinforcement learning, an agent is believed to find better solutions by updating actions based on the feedback received (18). Specifically, if an action yields more reward than expected, its value will increase. This learning process increases the likelihood of maximizing future rewards and is mediated by the exploration/exploitation trade-off (50, 51). Our results suggest that this learning process depends on accurate credit assignment, with random performance feedback clearly impairing this learning process. Interestingly, despite learning being impaired in the random feedback group, their performance was consistently better compared with all other groups (apart from Group-R+CF). This highlights that feedback that is paired with reward is potentially more motivating than either of the reward types independently even when the feedback is chosen at random. More specifically, performance improvements in Group-R+RF matched those of Group-R+CF over the first 50–75 trials across a range of variables (i.e., MT, FI, and vel_max/min_). Thereafter, improvements flatlined, whereas Group-R+CF continued to improve their performance. This suggests that, at least initially, random feedback presented as a score carried a motivational signal that led to performance improvements.

Experimental designs investigating the effects of reward on behavior predominantly rely on explicit reward types such as monetary incentive and performance-based feedback. However, in everyday life other forms of reward that are not necessarily financial in nature (i.e., recognition, prestige peer group approval) are equally present. Yet, how such nonfinancial reward types influence motor behavior and more specifically sequential motor behavior is less understood. For example, recent work using nonfinancial incentives found reduced adaptation rates during motor learning rather than improvements in performance as we found here (52, 53). Therefore, financial and nonfinancial reward types may influence motor behavior distinctively, which should be addressed in more depth in future research.

Combining monetary incentive with correct performance feedback not only maximized performance gains but also led to a greater amount of movement fusion. Fusion describes the process of blending together a series of distinct movements into a single continuous action. However, previous work using a simpler sequential reaching task showed that fusion takes up to 8 days (1,200–2,000 trials) (26, 27, 54). This highlights that movement fusion is characterized by a very slow learning process that is not simply the logical consequence of training. In contrast, participants in the present study showed clear fusion after a single training session (200 training trials), with this being maximized in Group_-R+CF_. Movement fusion was strongly associated not only with improvements in MT (via both increases in vel_max_ and reductions in dwell times) but also with increases in movement smoothness. Improvements in jerk/smoothness have been shown to reduce metabolic costs, thereby enhancing overall movement efficiency (31, 32). *Experiment 2* showed that performance in Group_-R+CF_ was smoother and exhibited greater similarity to a minimum-jerk trajectory through movement fusion. This suggests that movement fusion represents an effective strategy to perform faster, smoother, and more energetically efficient movements (26–28, 31, 32). Furthermore, *experiments 2* and *3* demonstrated that performance gains in Group_-R+CF_ were maintained across post-NR+NF and an additional testing day without either monetary incentive or performance feedback available. Similarly, albeit not significant, performance appeared to change the least across postassessments in Group_-R+CF_ in *experiment 1*. Additionally, the significant group effects show that Group_-R+CF_ performance was consistently better compared with the other groups. Taken together, these findings give rise to the interesting possibility that the transient effects of monetary incentives on performance can be maintained during periods without monetary incentive or performance feedback only if they were accompanied by improvements in kinematic efficiency (i.e., movement fusion). Alternatively, the long-term retention of performance gains may also be explained within the context of associative learning (55, 56). According to this framework, repetitive pairing of fast MTs with monetary incentive during training may induce an implicit association between two events that can remain even when reward is removed (55, 56). This in turn could account for the long-term retention of performance gains across an additional testing day.

Interestingly, we also observed gradual improvements in performance across variables for Group_-NR+NF_ in *experiment 2*. Despite them being markedly smaller compared with Group_-R+CF_, they suggest that performance improvements may also be driven by intrinsic rewards such as proprioceptive feedback, especially when explicit (external) rewards are lacking. This would fit with the small but steady improvements seen in FI for Group_-NR+NF_. Performing more continuous and smoother actions is linked to a reduction in metabolic costs (31, 32). Consequently, reducing that cost could be an intrinsic reward signal that guides future actions. However, it is important to note that such intrinsic reinforcement appears to be associated with shallower learning curves and therefore slower performance improvements as seen in previous research on movement fusion (26, 27).

Our results showed that movement fusion was associated with smoother and, with regard to minimizing jerk, more efficient execution. Interestingly, reaching movements performed by stroke patients exhibit reduced smoothness (57–60), with increases in jerk being due to a decomposition of movement into a series of submovements (57–60). However, over the course of the recovery process, performance becomes smoother as these submovements are progressively blended (57–60). Considering this theoretical proximity to the concept of movement fusion, we speculate that stroke recovery and movement fusion may follow similar principles. Consequently, fusion facilitated by monetary incentive in combination with performance feedback could be a powerful tool in stroke rehabilitation to promote smooth and efficient sequential actions that form an essential component of everyday life activities.

In summary, monetary incentive and performance feedback have dissociable effects on motor behavior. Importantly, pairing both maximized performance gains and accelerated the slow optimization process of movement fusion that leads to stable improvements in the speed and efficiency of sequential actions.

## SUPPLEMENTAL DATA

10.6084/m9.figshare.16831774.v1 Supplemental Figs. S1–S4: https://doi.org/10.6084/m9.figshare.16831774.v1

## GRANTS

S. Sporn, X. Chen, and J. M. Galea were supported by European Research Council Grant MotMotLearn 637488.

## DISCLOSURES

No conflicts of interest, financial or otherwise, are declared by the authors.

## AUTHOR CONTRIBUTIONS

S.S. and J.M.G. conceived and designed research; S.S. performed experiments; S.S. and X.C. analyzed data; S.S. and J.M.G. interpreted results of experiments; S.S. prepared figures; S.S. and J.M.G. drafted manuscript; S.S. and J.M.G. edited and revised manuscript; S.S., X.C., and J.M.G. approved final version of manuscript.

## ENDNOTE

At the request of the authors, readers are herein alerted to the fact that additional materials related to this manuscript may be found at https://osf.io/62wcz/. These materials are not a part of this manuscript and have not undergone peer review by the American Physiological Society (APS). APS and the journal editors take no responsibility for these materials, for the website address, or for any links to or from it.
